# Ability to understand and correctly follow HIV self‐test kit instructions for use: applying the cognitive interview technique in Malawi and Zambia

**DOI:** 10.1002/jia2.25253

**Published:** 2019-03-25

**Authors:** Musonda Simwinga, Moses K Kumwenda, Russell J Dacombe, Lusungu Kayira, Agness Muzumara, Cheryl C Johnson, Pitchaya Indravudh, Euphemia L Sibanda, Lot Nyirenda, Karin Hatzold, Elizabeth L Corbett, Helen Ayles, Miriam Taegtmeyer

**Affiliations:** ^1^ Zambart Lusaka Zambia; ^2^ Malawi Liverpool Wellcome Trust Clinical Research Programme Blantyre Malawi; ^3^ Department of International Public Health Liverpool School of Tropical Medicine Liverpool UK; ^4^ HIV and Global Hepatitis Department World Health Organization Geneva Switzerland; ^5^ Clinical Research Department Faculty of Infectious and Tropical Diseases London School of Hygiene and Tropical Medicine London UK; ^6^ Centre for Sexual Health HIV and AIDS Research Harare Zimbabwe; ^7^ Population Services International Johannesburg South Africa; ^8^ Department of Infectious Disease Epidemiology, London School of Hygiene and Tropical Medicine London UK

**Keywords:** HIV self‐test, performance, *in vitro* diagnosis, instructions for use, Zambia, Malawi

## Abstract

**Introduction:**

The ability to achieve an accurate test result and interpret it correctly is critical to the impact and effectiveness of HIV self‐testing (HIVST). Simple and easy‐to‐use devices, instructions for use (IFU) and other support tools have been shown to be key to good performance in sub‐Saharan Africa and may be highly contextual. The objective of this study was to explore the utility of cognitive interviewing in optimizing the local understanding of manufacturers’ IFUs to achieve an accurate HIVST result.

**Methods:**

Functionally literate and antiretroviral therapy‐naive participants were purposefully selected between May 2016 and June 2017 to represent intended users of HIV self‐tests from urban and rural areas in Malawi and Zambia. Participants were asked to follow IFUs for HIVST. We then conducted cognitive interviews and observed participants while they attempted to complete the HIVST steps using a structured guide, which mirrored the steps in the IFU. Qualitative data were analysed using a thematic approach.

**Results:**

Of a total of 61 participants, many successfully performed most steps in the IFU. Some had difficulties in understanding these and made errors, which could have led to incorrect test results, such as incorrect use of buffer and reading the results prematurely. Participants with lower levels of literacy and inexperience with standard pictorial images were more likely to struggle with IFUs. Difficulties tended to be more pronounced among those in rural settings. Ambiguous terms and translations in the IFU, unfamiliar images and symbols, and unclear order of the steps to be followed were most commonly linked to errors and lower comprehension among participants. Feedback was provided to the manufacturer on the findings, which resulted in further optimization of IFUs.

**Conclusions:**

Cognitive interviewing identifies local difficulties in conducting HIVST from manufacturer‐translated IFUs. It is a useful and practical methodology to optimize IFUs and make them more understandable.

## 1 Introduction

HIV self‐testing (HIVST) is increasingly being introduced as a testing approach recommended by the World Health Organization (WHO) to reach those who may not otherwise test 1, 2. Key advantages of HIVST are its high acceptability among men, young people and key populations, who often prefer the privacy and convenience of self‐testing over other HIV testing options 3. Without the ability to perform the test and interpret the results correctly, many of the potential benefits of HIVST are lost 4.

During development and for regulatory approvals, manufacturers provide the results of a process of evaluation that includes studies on ease of use and comprehension of test kit materials. An assessment of how the kit (device plus supporting materials) performs among untrained self‐testers is part of the standard regulatory approval process. Manufacturers’ pre‐submission enquiries 5 and full product dossiers undergo comprehensive assessment before site inspection and laboratory evaluations of performance are conducted. Approval implies that resource‐limited settings can have confidence that self‐use products have been rigorously evaluated 6. At the time of writing, one product has been prequalified by WHO with four approved for procurement with donor funds on an interim basis by the Global Fund's Expert Review Panel for Diagnostics 7.

The results of HIVST by untrained users have been shown to be relatively accurate though variable. Both oral fluid‐ and blood‐based HIVST have shown acceptable accuracy 8, especially when conducted with additional support in small‐scale assessments in sub‐Saharan Africa 9, 10, 11, 12, 13, 14, 15. External packaging, instructions for use (IFU) and any supplementary materials can impact the ability of users to correctly perform a self‐test and interpret the results. In Zimbabwe, overly wordy instructions were shown to result in poor outcomes in rural settings 15. In South Africa, poor self‐test outcomes were reported among healthcare workers who did not receive clear instructions on how to use and interpret the results of oral fluid‐based tests 13. A study comparing the usability of different prototypes of oral fluid‐ and blood‐based tests found participants confused by IFUs, even when the instructions had been specifically adapted for self‐test use 8. Video evaluation showed multiple errors in specimen collection, use of buffer, read times and interpretation of results, regardless of whether the kit was oral fluid based or blood based 16. Errors persisted even after self‐test prototypes were further adapted 9, 10, 12, 14, 17. Blood‐based self‐tests have been shown to be more sensitive than oral fluid‐based tests, but evidence suggests that invalid results among self‐testers may be also be higher 18, 19, 20, 21. Such variability presents a dilemma to potential implementers and to country regulatory authorities, and defeats the purpose of HIVST.

Cognitive interviewing has often been used to identify likely sources of response error in survey questionnaires. Using verbal probing to guide “thinking out loud,” it evaluates people's comprehension of specific words and phrases, assessing relevance and acceptability in a particular context 22, 23. We aimed to estimate the utility of cognitive interviewing in optimizing the local understanding of manufacturers’ IFUs to achieve an accurate HIVST result. To do this, we adapted cognitive interviewing techniques to include not only verbal comprehension but also in‐depth qualitative interviews and the observation of the ability to follow instructions. We tested the use of this adapted approach to cognitive interviewing for IFU optimization in two African countries with low literacy levels – Malawi and Zambia.

## 2 Methods

This study was nested within the Self‐Testing Africa (STAR) consortium, a large‐scale evaluation of HIVST in Malawi, Zambia and Zimbabwe 24. Before conducting the cognitive interviews, professional translators hired by the manufacturers had translated the IFUs into the local languages (Chichewa in Malawi, and Bemba, Nyanja and Tonga in Zambia). The translated IFUs are available at: https://www.psi.org/star-hiv-self-testing-africa/.

Participants were purposefully selected to represent intended users of HIV self‐tests. We included adult men and women aged ≥18 years; 44 participants in Malawi (May 2016 to June 2017) and 17 participants in Zambia (May to August 2016). They were recruited from primary health facilities when they presented for HIV testing, and were eligible for inclusion if they demonstrated functional literacy when asked to read a short text in the local language and self‐reported that they were HIV negative or of unknown status and were not on antiretroviral therapy (ART). Participants were from six communities – two rural and two urban communities in Malawi, and one rural and one urban community in Zambia. We included both rural and urban communities as literacy levels and comprehension of IFUs was likely to vary between these 25. Cognitive interviews were conducted with them; in Malawi, we used three iterations, with each stage informing further refinement and adaptation of IFUs; 20 participants used the first iteration, 12 used the second iteration and 12 used the third iteration. Changes made at each stage in the interactive process were communicated to the manufacturer through e‐mails. In Zambia, we additionally recruited participants who received an HIVST at their home to ensure that the context (e.g. lighting) in which HIVST was conducted was considered. In Zambia, one iteration of the IFU was used and evaluated by all participants, and suggested changes communicated to the manufacturer through email.

**Figure 1:**
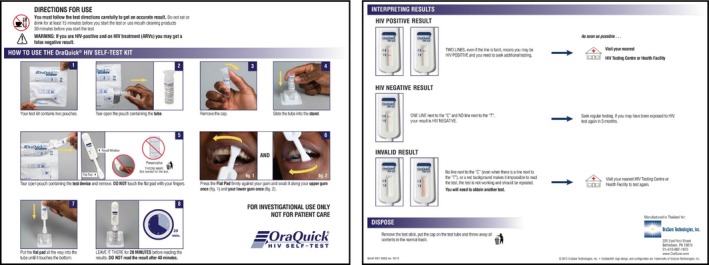
Manufacturer's original instructions for use. This figure is reproduced from OraQuick HIV Self‐Test instructions for use item number 3001‐XXXX rev.10/15 with permission from OraSure Technologies Inc.

Trained research assistants recruited participants. A structured guide that mirrored the steps depicted in the IFUs illustrated in Figure 1 informed the interviews. All participants were then given an OraQuick HIV Self‐Test kit, which contained this manufacturer's original IFU. They were asked to (1) read the instructions, (2) reflect on the pictorial and word instructions and explain these to the social scientist, (3) perform the actions depicted, and (4) reflect on how easy or difficult other members of their community would find the word and pictorial instructions. Scripted probes were included in the guide to ensure better understanding at each step, and research assistants were also trained to use spontaneous probes. Daily debriefing of field experiences was done to enhance the rigour of the cognitive interviewing process.

In Malawi, research staff took detailed observation notes at each step of the process. In Zambia, interviews were recorded, transcribed, translated into English and saved on password‐secured computers at the research offices. Data from both countries were analysed deductively and we used a thematic approach based on the various steps in the testing process. The comparative analysis presented here uses data from the second Malawian iteration and the single Zambian iteration, as these very closely matched the early feedback incorporated by the manufacturer from the first Malawian iteration. This involved researchers familiarizing themselves with the data, developing codes and then merging the codes into broader themes.

Readers should note that there were fewer steps in this manufacturer's original IFU than those in the final iteration included in our Table S1 that can be found in the supplementary materials submitted with this paper, because additional steps were added to the IFU as a result of early iterations.

### 2.1 Ethical considerations

In Malawi, we obtained ethics approvals from the College of Medicine Research Ethics Committee [Ref: P.01/16/1861] and the London School of Hygiene and Tropical Medicine (LSHTM) Ethics Committee [Ref: 10566]. In Zambia, the study was approved by the University of Zambia Biomedical Research Ethics Committee [Ref: 013‐11‐15] and LSHTM Ethics Committee [Ref: 10632]. All study participants provided informed consent.

## 3 Results

A total of 61 participants were included in this study. Over half of the participants in both countries understood the text and pictures used in the IFUs and could correctly conduct the self‐test and interpret the results. Performance errors, however, were identified at each of the 15 steps outlined in the manufacturer's original IFU, including unanticipated difficulties with opening the packet through to kit disposal. We present the results thematically and provide illustrative quotations. A summary table in the supplementary materials presents participants’ experiences at each step of following the IFU and compares results by country.

Over half of the participants found the textual and pictorial instructions to be complementary. Participants with lower literacy levels reported that the pictorial instructions improved their comprehension of the written instructions. When pictures were too difficult to understand, participants indicated that they used the textual instructions instead, such as the picture(s) in a step on when to start “timing the test.” Other factors that limited participants’ understanding and performance of certain instructions included translation errors, use of complicated terms, use of unfamiliar images and symbols, and the order in which the text and pictures describing the steps were presented.

### 3.1 Language (use of complicated terms) and low literacy

Translations and the use of complicated terms led to some misunderstanding of the IFUs and user errors. Sometimes these issues arose from translation errors in the IFUs themselves, and at other times it was lack of familiarity with certain terms. For example, some participants in Zambia found the translated word for “pouch” in the instructions too difficult to understand.

Two pouches? Someone would get confused, yes. At least put simpler words because someone would ask, “what are pouches?” and may be guess that these are pouches (female, 29 years, Kanakantapa, rural, Zambia).

The translation of “flat pad” in instruction 7 was also difficult for some people in Malawi and failure to understand the meaning of the local word resulted in a few participants touching the “flat pad” as illustrated by this quote: *Both sides are flat pads. The instruction should read, “gwirani kwakukuluko osati kwakung'ono” [touch the large side and not the smaller side] (female, 44 years, Madziabango, rural, Malawi)*. Participants also found words and phrases like “swab,” “press the pad firmly” confusing. This resulted in some participants doing odd things like pressing the pad hard “so that it could accumulate an adequate specimen” or placing the pad on their teeth and gums.

A related challenge was the variation in languages and dialects used within countries. Rural areas tended to use the original languages while urban areas tended to use colloquial versions. In Malawi, the Chichewa translation of the word gum was *usinini* but some participants from rural Malawi said this was a confusing translation and suggested *nkhama* instead. Thus, rural populations with poor literacy were more likely to struggle with understanding the messages in the IFUs and thereby made more errors. Rural participants who did comprehend IFUs and completed the self‐test correctly indicated that they relied on the pictorial instructions rather than the text.

### 3.2 Unfamiliar images and symbols

Images and pictorial illustrations in the IFU were meant to enhance understanding and performance of the instructions when used individually or complementarily with the word instructions. However, participants did not understand the meaning of some images that were not familiar to their local context. Over half the participants in both countries incorrectly interpreted the cutlery symbol in the directions at the top of the IFU (see Table S1) due to lack of familiarity with using cutlery for eating:

… the picture does not make sense. What does the cutlery mean? Use a toothbrush and Colgate and put in mind that most Malawians do not use forks [male, 20 years, Zingwangwa urban, Malawi].

Instead, participants from both countries interpreted the image to mean “avoid cutting oneself,” “do not eat or drink contents of the test‐kit” and “do not use a knife or fork to open the test kit.” The fact that over half of the participants performed this and other instructions successfully points to the fact that pictorial and word instructions complemented each other and common sense prevailed.

A red line was drawn through images/pictures to warn participants not to carry out certain actions such as not to pour out the liquid (instruction 5). However, over half the participants preferred crossed red lines as used in warning signs in Malawi and Zambia.

If this picture was like this (makes a gesture with crossed hands to make an X) it would show that you should not do this … (female, 35 years, Mtendere, urban, Zambia).

### 3.3 Presentation of images and instructions

Circumstances that made interpretation difficult included information that was clustered within an instruction. Instruction number one contained images of a wristwatch, a digital watch and a phone to illustrate the importance of having a timing device. Some participants felt that having several images talking about one thing was misleading: *It is not clear. Is it a time or a date? (male, 29 years, Limbe, urban, Malawi)*. Indeed, over half the participants did not have timing devices; a potential challenge for ensuring correct reading times even if the instruction was clearly understood.

Different font types, sizes and colours also created confusion. Some participants observed that the instruction about removing the test device from the pouch was written in a small font and therefore difficult to see. Other participants said that the presentation of instructions in different font sizes and colours could prompt users to think that the instructions in question were not important.

Some word instructions did not have corresponding pictorial instructions and vice versa. For instance, the written statement that users should not use “mouth cleaning products 30 minutes before you start the test” did not have a corresponding picture/image. This affected the participants’ understanding of the instructions.

### 3.4 Order, clarity and adequacy of instructions/messages

The order or positioning of instructions was critical to avoid confusion. The red capitalized instruction “IF YOU READ BEFORE 20 MINUTES, RESULTS MAY NOT BE CORRECT” came after the user had tested and had been told to “leave the test device in the tube for 20 minutes before reading the results” without telling the user the implications of reading the results earlier than 20 minutes or after 40 minutes. According to the participants, presenting the implications earlier could have enhanced understanding of and adherence to instructions.

Inadequate information was also a source of poor cognition of the instructions. For example, a warning that being on ART may lead to incorrect (false‐negative) results was included, because retesting to confirm a previously known positive status has previously been reported and can lead to a false‐negative self‐test result 15, 26, 27. However, participants found this message to be confusing and did not understand how someone who is infected with HIV could obtain negative results when the intention of the test‐kit was to detect HIV: *How does one that is HIV positive get negative results? (female, 22 years, Mpemba, rural, Malawi). I don't understand, how can you get a false‐negative result? (female, 29 years, Kanakantapa, rural, Zambia)*.

## 4 Discussion

Our study aimed to evaluate the utility of cognitive interviewing in optimizing the local understanding of manufacturers’ IFUs to achieve an accurate HIVST result. This study was nested within the STAR consortium, a large‐scale evaluation of HIVST in Malawi, Zambia and Zimbabwe. We used cognitive interviewing in Malawi and Zambia, two African countries with low literacy levels, to rapidly identify how well users of oral fluid‐based HIV self‐test kits were able to understand IFUs and their ability to obtain accurate results.

The use of cognitive interviewing in the iterative creation and improvement of questionnaires and health promotion materials has been described elsewhere, particularly for exploring how survey questions are understood by research participants and how these require significant contextual adaptation 28, 29, 30. Results from cognitive interviews often show that some survey questions are appropriately interpreted by respondents, and others show significant differences between what the researchers intended them to measure and what they actually do 31, 32. We found the same with IFU with some instructions being easy to understand and conduct as intended by the manufacturers and others not. We adapted these methods by combining the step‐by‐step drill down on each IFU instruction with qualitative data capture and observed the errors. This allowed us to gain additional insights on how to best tailor support materials, which was not possible from less targeted interviews, even when supported by video observation 16. We found the principle of cognitive interviewing to be an essential element, i.e. taking time to explore the understanding of each instruction, statement or question.

While systematic reviews and evaluations have shown that HIVST can be successfully conducted by the intended users without in‐person demonstrations 8, we feel that additional support materials such as checklists, videos and in‐person demonstrations are likely to be particularly important for rural and urban populations with low literacy 33, 34, 35. Viewing a demonstration video increased adolescents’ and adults’ confidence in their ability to self‐test in Zambia 36. Providing an in‐person demonstration resulted in high sensitivity of oral fluid self‐testing in KwaZulu Natal 17, which did not happen when the oral fluid test was conducted unsupervised in similar settings 12. Demonstrations of how to use the kit are an integral part of our studies in Malawi, Zambia and Zimbabwe 10, 11, 37, 38. Demonstration materials do, however, need to be tailored to the context. Men who have sex with men in South Africa preferred fingerstick self‐testing but were better able to perform the oral fluid tests resulting in the need for additional instructional resources for blood‐based testing in this context 39. Ortblad et al. reported that when peer educators working with female sex workers in Uganda gave an HIVST demonstration based on materials developed without detailed knowledge of common misunderstandings, the sex workers struggled to correctly interpret the test results 40. In Zimbabwe, on the other hand, where female sex workers were shown a video based on findings from cognitive interviews, they were able to correctly perform and interpret oral fluid‐based HIVST 38. Training lay people, including women with untested male partners, on how to demonstrate a self‐test may be another option. In Malawi and Kenya, HIVST delivered to the male partners of pregnant women resulted in high uptake and increased couples’ testing 41, 42, 43. Over time, as knowledge and awareness of HIVST increases, the need for cognitive interviewing is likely to decline.

Our findings were used to provide feedback to the manufacturer and resulted in further optimization of IFUs as well as the development of demonstrations used in the STAR project to further improve performance. However, iterations and adaptation of the actual IFUs are neither possible nor desirable for every community, every key population or subgroup. They are also not possible from a regulatory point of view, since regulators and WHO prequalification regard only the IFUs in the final prequalified product as approved package inserts, allowing only simple changes for clarification and translation 44. The onus is thus on programmes to ensure appropriate introduction of HIVST. A practical toolkit aimed at programmes wishing to introduce HIVST is now being developed to provide further guidance on how to optimize HIVST implementation, including guidance on how to conduct and interpret findings from cognitive interviews exploring IFUs.

Our study had several limitations, such as different data collection techniques. Data collection in Malawi was captured using an observational checklist while a digital audio recorder was used in Zambia. However, the differences in data capturing techniques did little to influence our analysis, since the focus of data synthesis was on how each client understood each instruction, and how they practically translated word and pictorial instructions when performing an HIVST. Malawi conducted three iterations with the aim of improving the IFUs at each iterative stage while Zambia only had a single iteration and fewer participants, and this might have influenced our findings, although the comparison used the same IFU iteration and the same principles of cognitive interviewing methods. Professional translations done by the manufacturers had several problems and required revisions by researchers within the study team in both countries. Finally, similar cognitive interviews conducted in Zimbabwe a year before this study informed the development of study tools in Malawi and Zambia; however, as the team iteratively changed the IFUs several times, there was no version that exactly matched the ones used in Malawi and Zambia. We felt that the methods did not overlap sufficiently to include them, limiting the potential for comparison in a third context.

## 5 Conclusions

Cognitive interviewing provided an excellent methodological approach to assessing IFU but required some adaptation to include direct observation of test performance. The adapted cognitive methodology we used highlighted several errors that were common across both countries and helped us to determine the nature of support users might need and to pre‐empt common test performance problems, through improved translations and adaptation of manufacturers’ IFUs. Efforts to further optimize performance may not always be feasible through IFUs alone but may require the addition of demonstrations and support tools in settings and populations with low education and literacy levels.

## Competing interests

No potential conflict of interest is reported by the authors.

## Authors’ contributions

MS and MK wrote first draft and led the entire writing process. LK and AM were the principal data collectors and contributed to the first draft. PI, ES, LN and RD also contributed to first draft and reviewed subsequent iterations. CJ, KH, ELC and HA provided a thorough review of the drafts and contributed to the second draft. MT contributed to all the iterations and provided technical guidance to MS and MK.

## Supporting information


**Table S1:** Results (participants’ experiences).

## References

[1] World Health Organization . Guidelines on HIV self‐testing and partner notification: supplement to consolidated guidelines on HIV testing services. Geneva, Switzerland: World Health Organization; 2016. 27977094

[2] Johnson C , Baggaley R , Forsythe S , van Rooyen H , Ford N , Napierala Mavedzenge S , et al. Realizing the potential for HIV self‐testing. AIDS Behav. 2014;18 Suppl 4:S391–5. 10.1007/s10461-014-0832-x. 24986599

[3] Indravudh PP , Sibanda EL , d'Elbee M , Kumwenda MK , Ringwald B , Maringwa G , et al. “I will choose when to test, where I want to test”: investigating young people's preferences for HIV self‐testing in Malawi and Zimbabwe. AIDS. 2017;31(Suppl 3):S203–12. 10.1097/qad.0000000000001516 28665878 PMC5497773

[4] Shanks L , Klarkowski D , O'Brien DP . False positive HIV diagnoses in resource limited settings: operational lessons learned for HIV programmes. PLoS ONE. 2013;8(3):e59906 10.1371/journal.pone.0059906. 23527284 PMC3603939

[5] World Health Organization . Technical specifications series for submission to WHO prequalification – diagnostic assessment: human immunodeficiency virus (HIV) rapid diagnostic tests for professional use and/or self‐testing. Geneva: World Health Organization; 2016 Licence: CC BY‐NC‐SA 3.0 IGO. 26 p.

[6] World Health Organization . WHO prequalification of in vitro diagnostics public report. Product: OraQuick HIV self‐test. WHO reference number: PQDx 0159‐055‐01 (version 2.0). Geneva, Switzerland: World Health Organization; 2017 [cited 2018 Mar 10]. Available from: http://www.who.int/diagnostics_laboratory/evaluations/pq-list/170720_final_amended_pqdx_0159_055_01_oraquick_hiv_self_test_v2.pdf

[7] The Global Fund to Fight AIDS, Tuberculosis and Malaria . List of HIV diagnostic test kits and equipments classified according to the Global Fund Quality Assurance Policy (version 17). Geneva, Switzerland: The Global Fund; 2017 [cited 2018 Mar 10]. Available from: https://www.theglobalfund.org/media/5878/psm_productshiv-who_list_en.pdf

[8] Figueroa C , Johnson C , Ford N , Sands A , Dalal S , Meurant R , et al. Reliability of HIV rapid diagnostic tests for self‐testing compared with testing by health‐care workers: a systematic review and meta‐analysis. Lancet HIV. 2018;5(6):e277–90. 10.1016/s2352-3018(18)30044-4. 29703707 PMC5986793

[9] Asiimwe S , Oloya J , Song X , Whalen CC . Accuracy of un‐supervised versus provider‐supervised self‐administered HIV testing in Uganda: a randomized implementation trial. AIDS Behav. 2014;18(12):2477–84. 10.1007/s10461-014-0765-4. 24691923 PMC4183743

[10] Choko AT , Desmond N , Webb EL , Chavula K , Napierala‐Mavedzenge S , Gaydos CA , et al. The uptake and accuracy of oral kits for HIV self‐testing in high HIV prevalence setting: a cross‐sectional feasibility study in Blantyre, Malawi. PLoS Med. 2011;8(10):e1001102 10.1371/journal.pmed.1001102. 21990966 PMC3186813

[11] Choko AT , MacPherson P , Webb EL , Willey BA , Feasy H , Sambakunsi R , et al. Uptake, accuracy, safety, and linkage into care over two years of promoting annual self‐testing for HIV in Blantyre, Malawi: a community‐based prospective study. PLoS Med. 2015;12(9):e1001873 10.1371/journal.pmed.1001873. 26348035 PMC4562710

[12] Kurth AE , Cleland CM , Chhun N , Sidle JE , Were E , Naanyu V , et al. Accuracy and acceptability of oral fluid HIV self‐testing in a general adult population in Kenya. AIDS Behav. 2016;20(4):870–9. 10.1007/s10461-015-1213-9. 26438487 PMC4799243

[13] Pant Pai N , Behlim T , Abrahams L , Vadnais C , Shivkumar S , Pillay S , et al. Will an unsupervised self‐testing strategy for HIV work in health care workers of South Africa? A cross sectional pilot feasibility study. PLoS ONE. 2013;8(11):e79772 10.1371/journal.pone.0079772. 24312185 PMC3842310

[14] Smith P , Wallace M , Bekker LG . Adolescents’ experience of a rapid HIV self‐testing device in youth‐friendly clinic settings in Cape Town South Africa: a cross‐sectional community based usability study. J Int AIDS Soc. 2016;19(1):21111 10.7448/ias.19.1.21111. 28406597 PMC5380981

[15] Mavedzenge SN , Sibanda E , Mavengere Y , Hatzold K , Mugurungi O , Ncube G , et al. Supervised HIV self‐testing to inform implementation and scale up of self‐testing in Zimbabwe. 8th IAS Conference on HIV Pathogenesis, Treatment and Prevention (IAS 2015); 2015 Jul 19‐22; Vancouver. [cited 2018 May 4]. Available from: http://hivst.org/tools/2016120supervised-hiv-self-testing-to-inform-implementation-and-scale-up-of-self-testing-in-zimbabwe

[16] Peck RB , Lim JM , van Rooyen H , Mukoma W , Chepuka L , Bansil P , et al. What should the ideal HIV self‐test look like? A usability study of test prototypes in unsupervised HIV self‐testing in Kenya, Malawi, and South Africa. AIDS Behav. 2014;18 Suppl 4:S422–32. 10.1007/s10461-014-0818-8. 24947852

[17] Martinez Perez G , Steele SJ , Govender I , Arellano G , Mkwamba A , Hadebe M , et al. Supervised oral HIV self‐testing is accurate in rural KwaZulu‐Natal, South Africa. Trop Med Int Health. 2016;21(6):759–67. 10.1111/tmi.12703. 27098272

[18] Gaydos CA , Hsieh Y‐H , Harvey L , Burah A , Won H , Jett‐Goheen M , et al. Will patients “opt in” to perform their own rapid HIV test in the emergency department? Ann Emerg Med. 2011;58(1 Suppl 1):S74–8. 10.1016/j.annemergmed.2011.03.029. 21684413 PMC3187596

[19] Ibitoye M , Frasca T , Giguere R , Carballo‐Diéguez A . Home testing past, present and future: lessons learned and implications for HIV home tests. AIDS Behav. 2014;18(5):933 10.1007/s10461-013-0668-9. 24281697 PMC3988264

[20] Lee VJ , Tan SC , Earnest A , Seong PS , Tan HH , Leo YS . User acceptability and feasibility of self‐testing with HIV rapid tests. J Acquir Immune Defic Syndr. 2007;45(4):449–53. 10.1097/QAI.0b013e318095a3f3. 17554213

[21] de la Fuente L , Rosales‐Statkus ME , Hoyos J , Pulido J , Santos S , Bravo MJ , et al. Are participants in a street‐based HIV testing program able to perform their own rapid test and interpret the results? PLoS ONE. 2012;7(10):e46555 10.1371/journal.pone.0046555. 23056342 PMC3466298

[22] Willis GB . Cognitive interviewing: a “how to” guide, from the short course “reducing survey error through research on the cognitive and decision processes in surveys.”Meeting of the American Statistical Association, Dallas (TX); 1999.

[23] Willis GB , Artino AR Jr . What do our respondents think we're asking? Using cognitive interviewing to improve medical education surveys. J Grad Med Educ. 2013;5(3):353–6. 10.4300/jgme-d-13-00154.1. 24404294 PMC3771159

[24] HIV Self‐Testing Africa . The STAR Initiative [Internet]. Washington, DC: PSI [cited 2018 Dec 28]. Available from: https://www.eatright.org/]; https://www.psi.org/star-hiv-self-testing-africa

[25] Zhang Y . Urban‐rural literacy gaps in Sub‐Saharan Africa: the roles of socioeconomic status and school quality. Comp Educ Rev. 2006;50(4):581–602. 10.1086/507056.

[26] Johnson CC , Sands A , Urassa W , Baggaley R . Alert, but not alarmed – a comment on “Towards more accurate HIV testing in sub‐Saharan Africa: a multi‐site evaluation of HIV RDTs and risk factors for false positives.” J Int AIDS Soc. 2017;20(1):22042 10.7448/ias.20.1.22042. 28664683 PMC5515062

[27] Moore A , Cassidi T , Steele SJ , Shroufi A , Ntuli N , Ndani L , et al. Self‐testing: an effective means of increasing HIV‐testing and status awareness. 9th IAS Conference on HIV Science (IAS 2017); 2017 July 23‐27; Paris, France [cited 2018 Mar 10]. Available from: http://www.ias2017.org/Portals/1/Files/IAS2017_LO.compressed.pdf

[28] Egger‐Rainer A . Enhancing validity through cognitive interviewing. A methodological example using the Epilepsy Monitoring Unit Comfort Questionnaire (EMUCQ). J Adv Nurs. 2019;75(1):224–33. 10.1111/jan.13867. 30289559 PMC7379296

[29] Vreeman RC , Nyandiko WM , Ayaya SO , Walumbe EG , Inui TS . Cognitive interviewing for cross‐cultural adaptation of pediatric antiretroviral therapy adherence measurement items. Int J Behav Med. 2014;21(1):186–96. 10.1007/s12529-012-9283-9. 23188670

[30] Drennan J . Cognitive interviewing: verbal data in the design and pretesting of questionnaires. J Adv Nurs. 2003;42(1):57–63. 10.1046/j.1365-2648.2003.02579.x. 12641812

[31] De Silva MJ , Harpham T , Tuan T , Bartolini R , Penny ME , Huttly SR . Psychometric and cognitive validation of a social capital measurement tool in Peru and Vietnam. Soc Sci Med. 2006;62(4):941–53. 10.1016/j.socscimed.2005.06.050. 16095787

[32] Bowden A , Fox‐Rushby JA , Nyandieka L , Wanjau J . Methods for pre‐testing and piloting survey questions: illustrations from the KENQOL survey of health‐related quality of life. Health Policy Plan. 2002;17(3):322–30. 10.1093/heapol/17.3.322. 12135999

[33] Tonen‐Wolyec S , Batina‐Agasa S , Muwonga J , N'kulu F , Bouassa RSM , Belec L . Evaluation of the practicability and virological performance of finger‐stick whole‐blood HIV self‐testing in French‐speaking sub‐Saharan Africa. PLoS ONE. 2018;13(1):e0189475 10.1371/journal.pone.0189475. 29320504 PMC5761859

[34] Tonen‐Wolyec S , Mboup S , Gresenguet G , Bouassa RB , Belec L . Insufficient education is a challenge for HIV self‐testing. Lancet HIV. 2018;5(7):e341 10.1016/s2352-3018(18)30141-3. 30052505

[35] Wei C , Yan L , Li J , Su X , Lippman S , Yan H . Which user errors matter during HIV self‐testing? A qualitative participant observation study of men who have sex with men (MSM) in China. BMC Public Health. 2018;18(1):1108 10.1186/s12889-018-6007-3. 30200905 PMC6131779

[36] Zanolini A , Chipungu J , Vinikoor MJ , Bosomprah S , Mafwenko M , Holmes CB , et al. HIV self‐testing in Lusaka Province, Zambia: acceptability, comprehension of testing instructions, and individual preferences for self‐test kit distribution in a population‐based sample of adolescents and adults. AIDS Res Hum Retroviruses. 2017;34(3):254–60. 10.1089/aid.2017.0156. 28969432 PMC5863088

[37] Kapaku KN , Neuman M , Maluzi K , Sigande L , Nalubamba M , Taegtmeyer M , et al. Is OraQuick^®^HIV‐self‐testing valid among intended users? Analysis from a clinical performance study in Lusaka, Zambia. 9th IAS Conference on HIV Science (IAS 2017); 2017 July 23‐27; Paris, France [cited 2018 May 4]. Available from: http://www.ias2017.org/Portals/1/Files/IAS2017_LO.compressed.pdf

[38] Sibanda EL . Usability and validity of oral fluid self‐tests among intended users: experiences from Malawi, Zambia and Zimbabwe. 9th IAS Conference on HIV Science (IAS 2017); 2017 July 23‐27; Paris, France [cited 2018 May 4]. Available from: http://programme.ias2017.org/Programme/Session/76

[39] Lippman SA , Gilmore HJ , Lane T , Radebe O , Chen Y‐H , Mlotshwa N , et al. Ability to use oral fluid and fingerstick HIV self‐testing (HIVST) among South African MSM. PLoS ONE. 2018;13(11):e0206849 10.1371/journal.pone.0206849. 30408055 PMC6224086

[40] Ortblad KF , Musoke DK , Ngabirano T , Nakitende A , Haberer JE , McConnell M , et al. Female sex workers often incorrectly interpret HIV self‐test results in Uganda. J Acquir Immune Defic Syndr. 2018;79(1):e42–5. 10.1097/QAI.0000000000001765. 29847478 PMC6095458

[41] Masters SH , Agot K , Obonyo B , Napierala Mavedzenge S , Maman S , Thirumurthy H . Promoting partner testing and couples testing through secondary distribution of HIV self‐tests: a randomized clinical trial. PLoS Med. 2016;13(11):e1002166 10.1371/journal.pmed.1002166. 27824882 PMC5100966

[42] Gichangi A , Wambua J , Gohole A , Mutwiwa S , Njogu R , Bazant E , et al. Provision of oral HIV self‐test kits triples uptake of HIV testing among male partners of antenatal care clients: results of a randomized trial in Kenya. 21st International Aids Conference (AIDS 2016); 2016 Jul 18‐22; Durban, South Africa; 2016 [cited 2018 May 4]. Available from: http://www.aids2016.org/Portals/0/File/AIDS2016_Abstracts_LOW.pdf

[43] Choko AT , Kumwenda MK , Johnson CC , Sakala DW , Chikalipo MC , Fielding K , et al. Acceptability of woman‐delivered HIV self‐testing to the male partner, and additional interventions: a qualitative study of antenatal care participants in Malawi. J Int AIDS Soc. 2017;20(1):21610 10.7448/ias.20.1.21610. 28691442 PMC5515040

[44] World Health Organization . Reportable changes to a WHO prequalified in vitro diagnostic medical device. Geneva, Switzerland: World Health Organization; 2016.

