# Supplementary material for: Ability to understand and correctly follow HIV self‐test kit instructions for use: applying the cognitive interview technique in Malawi and Zambia
Source: J Int AIDS Soc. 2019 Mar 25;22(Suppl Suppl 1):e25253. doi: 10.1002/jia2.25253 (PMC6432102; doi:10.1002/jia2.25253)
Supplement: Supplementary file 1 — Table S1: Results (participants’ experiences). [file JIA2-22-e25253-s002.docx]

**Table 1: Results (participants’ experiences)**

| **Step/ Instruction** | **Participant Understanding** | | **Implications for IFUs and support materials** |
| --- | --- | --- | --- |
|  | **Zambia** | **Malawi** |  |
| Heading information and Warning | 1. All participants correctly construed the lay out of the IFU pointing out what were the contents of the front and back of the IFU. | 1. Only 2/12 participants were confused about the layout partly because the front and back pages of the IFU were not numbered. | Both the written and pictorial instructions need to be very clear with the former succinctly describing the latter. |
|  | 1. Participants often required some time to understand the layout and order of instructions. | 1. Participants often required some time to understand the layout and order of instructions. |  |
|  | 1. Pictorial instructions were very useful to participants who did not have enough time to read all written instructions and the semi illiterate. | 1. Pictorial instructions were very useful to the semi illiterate; they were generally very clear. |  |
| **Step/ Instruction** | **Participant Understanding** | | **Implications for IFUs and support materials** |
|  | **Zambia** | **Malawi** |  |
| Direction for use:  (Interpretation of picture of cutlery and Warning (not to use kit if HIV positive and on treatment)  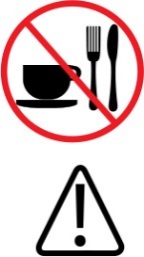 | 1. Participants said Instructions about when to test after eating food were clear. | - 1. While instructions about when to test after eating food were clear, one participant said the reasons were not explained. | 1. Pictures used in the IFU should be large, very clear and relate to the local context and everyday life. Zambians and Malawians eat using hands not knives and folks. 2. Both countries suggested use of big red X for the ‘do not’ sign- a common road signage to replace the circle with line.. 3. Warning on testing when on ART: the instructions need to be clear and even targeted. This instruction should simply tell those who are positive not to test using the kit. 4. Written and pictorial instructions when used together enhances understanding by the self-tester. |
|  | 1. Over half the participants incorrectly interpreted the cutlery picture, e.g. as a warning to be careful not to cut themselves; a warning not to use knife and fork to open kit; a warning not to eat the kit’s contents. Others mistook picture for something else e.g. knife for a comb. | - 1. The cutlery picture was clear but not its meaning. They suggested use of things that Malawians would easily relate to. |  |
|  | 1. Warning: most participants (14/17) understood the warning. | - 1. Warning: four participants felt that the instruction was clear while another 4/12 participants said it was unclear. They wondered how a positive individual may get negative results. One participant felt that the instruction was not necessary as it was difficult for the illiterate to understand. |  |
| **Step/ Instruction** | **Participant Understanding** | | **Implications for IFUs and support materials** |
|  | **Zambia** | **Malawi** |  |
| Instruction 1: Way of timing the test  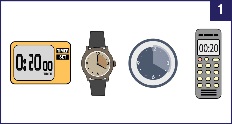 | 1. Most participants (16/17) correctly understood the instruction to mean that they needed a device to time the test. | 1. Half of the participants (6/12) wrongly understood the instruction to mean that they needed to assign some time to test and not a test device. | Lack of having a timing device was more likely to result to early interpretation of results.   1. The instruction should be very clear 1) that the tester needs a device such as these to time their test 2) that timing/ use of these devices will be done later. 2. Remove the phone; possibly remain with the watch. |
|  | 1. Most participants used a phone to time their tests | 1. Some participants especially in rural areas had difficulties accessing a device to time the test. |  |
|  | 1. Some participants thought that instruction 1 was not part of other instructions because the words were written in different font size and colour. | 1. Having more than one picture in the image was also confusing. According to them, one watch would suffice. |  |
|  |  | 1. The phone was often misinterpreted as a calculator. |  |
|  | 1. Some participants wanted to commence timing from this stage and sought guidance from the interviewers. |  |  |
|  |  | 1. The numbers on the digital watch were confusing. Some participants could not tell if this was a date or time. |  |
| **Step/ Instruction** | **Participant Understanding** | | **Implications for IFUs and support materials** |
|  | **Zambia** | **Malawi** |  |
| Instruction 2: Two pouches  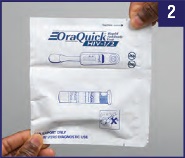 | 1. Some participants found the word ‘pouch’ difficult to understand. They contended that it will be even much more difficult for the ‘ordinary person’ to understand the word. | 1. Three (3/12) participants did not clearly understand the instruction until prompted by the researchers. | 1. Participants in Zambia suggested the words ‘pockets’ and ‘packets; in place of ‘pouches’. 2. Participants in Malawi suggested brighter pictures to accommodate those with sight problems. |
|  |  | 1. Most participants (9/12) easily understood and performed the instruction; they did not have problems to identify the 2 pouches. |  |
| Instruction 3: Removal of tube from the pouch  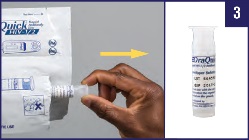 | 1. Most participants understood and performed the instructions but some did not see the provision for tearing open the pouch and so used different means: their nails, and teeth. | 1. Most participants understood and performed the instructions and performed it without challenges using both visualisation and touching. | 1. Increase font size for the instruction ‘Tear Here’. 2. Put a scissors sign on the instruction on both inner and outer packets. |
|  | 1. One participant opened the pouch containing the test device first. | 1. One participant did not remove the bottle from the package. |  |
|  |  | 1. Two individuals used the picture on the pouch to determine the side where the bottle was located. |  |
| **Step/ Instruction** | **Participant Understanding** | | **Implications for IFUs and support materials** |
|  | **Zambia** | **Malawi** |  |
| Instruction 4: Opening of the tube  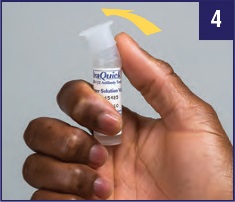 | 1. All participants understood the instruction and successfully opened the tube without spilling the contents. | 1. Most participants understood the instruction and successfully opened the tube. | 1. Prior knowledge about the liquid contents of the tube is important. 2. Indicate how the tube should be opened. |
|  | 1. Some thought that they should have been forewarned that there is a liquid in the tube so that they could be much more careful not to spill it. |  |  |
|  |  | 1. Three participants attempted to open the bottle in a screwing motion while one participant complained that *‘the instruction does not say how you should open it [Male, 36 years, Mpemba].* |  |
| Instruction 5: Use of the tube - not pouring out the liquid  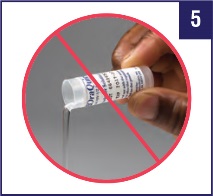 | 1. Most (15/17) participants understood the instruction as meaning not to pour out the liquid although two understood the opposite. | 1. Most (10/12) participants understood the instruction as meaning not to pour out the liquid. Like in Zambia, two understood the opposite. | Pouring out all the liquid content of the tube was more likely to result into invalid results.   1. Some participants suggested use of X as in the ‘do not’ signs 2. Picture should not show liquid being poured out (Malawi) |
|  |  | 1. Two participants did not understand the picture instruction but understood the word instruction and successfully performed the instruction. |  |
| **Step/ Instruction** | **Participant Understanding** | | **Implications for IFUs and support materials** |
|  | **Zambia** | **Malawi** |  |
| Instruction 6: Sliding the tube into the stand  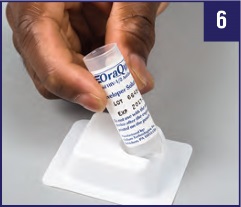 | 1. Although most participants managed to slide the tube into the stand, this was done with varying difficulty; most participants pushed the tube into the stand rather than slide it | 1. Although most participants said the instruction was clear, they nonetheless felt that inserting the tube onto the stand in a tilted position was hard. | 1. Most people are right handed so the picture should be repositioned. 2. Instruct participants to slide tube from the open end. 3. Emphasize the importance of applying reasonable pressure to insert the tube in the stand. |
|  | 1. One participant put the tube upside down and only put it in correct position at the time of inserting the test device in the tube. | 1. Some participants did not know that they needed to apply a bit of pressure. |  |
| Instruction 7: Removal of test device from pouch | 1. Most participants said the images were too clustered and the font size of the words too small even though all opened without touching the pad. | 1. Most participants said the images were too many and this undermined their cognition. However, half the participants said the written instructions were clear and correctly performed them. | 1. Segment the instructions into two or three parts. |
|  | 1. 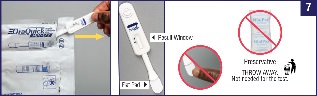Some participants did not see the preservative until later and did not know its use. | 1. One participant did not know the use of the preservative and another thought some people may understand the instruction on throwing away the preservative. |  |
| **Step/ Instruction** | **Participant Understanding** | | **Implications for IFUs and support materials** |
|  | **Zambia** | **Malawi** |  |
| Instruction 7: Removal of test device from pouch (continued) |  | 1. Translation of flat pad was difficult as both parts were seen as flat; 1 participant did not understand instruction regarding not touching the flat pad; 1 actually touched it. |  |
| Instruction 8: Collecting the specimen  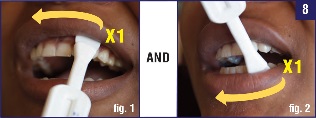 | 1. Although most participants said the instructions were clear, some did not do the swabbing properly; some just placed the test device on the upper gum, waited for some time, and then did the same to the lower gum. | 1. Most participants understood and performed the instructions properly. | Failure to collect a sufficient specimen was more likely to result into invalid results.   1. Change instruction from swab to ‘slide pad along. ‘ 2. If possible show picture of a hand holding the test device. 3. Improve the translation of some words. 4. Emphasize that the flat pad should be pressed gently in the gums and a bigger surface area of the flat pad should be in contact with the gums. |
|  | 1. Words and phrases like ‘swab’, ‘press firmly’ were said to be misleading. | 1. Some participants suggested a better translation of Chichewa word from usinini which means gums to nkhama which was more familiar. |  |
|  | 1. Some participants thought that it is the test device that should be pressed hard using fingers for it to suck as much specimen as possible; one placed the device on the upper gum only, another on upper and lower teeth alternately. | 1. One participant did not put the whole flat pad in the mouth afraid that it was hard; another placed the pad against the teeth and later wiped it with hands. |  |
|  |  | 1. Two participants were scared swabbing would hurt their gums; another thought that the flat pad was hard to move on a soft tissue |  |
| **Step/ Instruction** | **Participant Understanding** | | **Implications for IFUs and support materials** |
|  | **Zambia** | **Malawi** |  |
| Instruction 9: Placing the test device  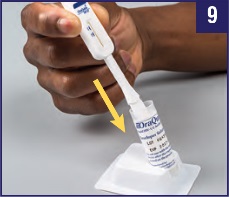 | 1. All, except one (16/17) participant, understood and carried out this instruction correctly. | 1. All participants (12/12) understood and carried out this instruction correctly. | None |
| Instruction 10: Timing of the test  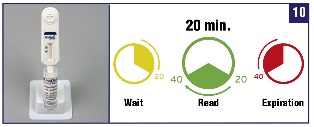 | 1. The written instructions were clear and easy to follow. However, the pictorial instructions were not; most participants relied on the written instructions to perform this step. | 1. Like in Zambia, written instructions were very clear and easy to follow. However, the pictorial instructions for this instruction was not. Thus, most participants depended on the written instructions to perform this step. | Limiting number of ‘clocks’ to two (for 20 and 40 minutes with the latter crossed) would reduce the confusion. |
|  | 1. One participant thought she had to wait 20 minutes before removing the device from the tube, and wait a further 20 minutes to read the results. | 1. Some participants incorrectly interpreted the word ‘expiration’ to mean the kit would expire in the indicated time. |  |
| **Step/ Instruction** | **Participant Understanding** | | **Implications for IFUs and support materials** |
|  | **Zambia** | **Malawi** |  |
| a. Interpreting results: HIV positive results  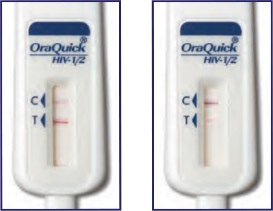 | 1. Both the written and pictorial instructions were clear and easy to follow but some participants observed that the faint line may not be seen by people with poor sight. | 1. Both the written and pictorial instructions were clear and easy to follow. | 1. Lines generated by folding of the instruction page contributed to the inability of participants to see link between the main and follow up instructions. 2. Move follow up information to under the image/ main instruction or extend the length of the arrow to clearly link the two. |
|  | 1. Some participants did not see the follow up instructions. | 1. Some participants did not see the follow up instructions. |  |
| b. Interpreting results: HIV negative results  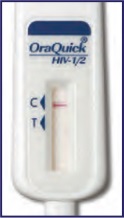 | 1. Both the written and pictorial instructions were clear and easy to follow. | 1. Both the written and pictorial instructions were clear and easy to follow. | Instruction on timing of reading the test results should come earlier. |
|  | 1. Some participants did not see the follow up instructions. | 1. Fewer participants than in previous step did not see follow up instructions- leant from previous step. |  |
|  | 1. Participants thought that instruction on not to read results before 20 minutes should have come earlier in the IFU. | 1. Participants thought that instruction on not to read results before 20 minutes should have come earlier in the IFU. |  |
| **Step/ Instruction** | **Participant Understanding** | | **Implications for IFUs and support materials** |
|  | **Zambia** | **Malawi** |  |
| c. Interpreting results: Invalid results  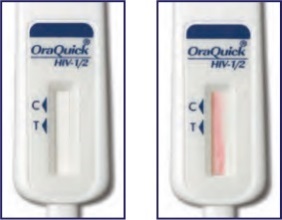 | 1. All but one (16/17) participant understood what an invalid result looks like. | 1. All participants understood what an invalid result looks like. | Ensure there are images to match the description/ instruction provided. |
|  | 1. Participants observed that there was no image for the description ‘no line next to the C, even where there is a line next to the T’, and no description for the blank image. | 1. One participant critically analysed the pictures saying that there was no picture that showed only one line on letter ‘T’. |  |
| Disposal  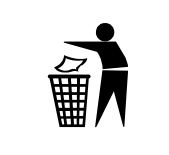 | 1. Some participants said this step seemed less important because the image was very small and some ignored the step altogether. | 1. Most participants understood the written and pictorial instructions. One felt that the picture is universal and easy to understand. | Enlarge the image. |
| Reading/Interpreting own results | 1. All participants did not have problems reading their HVST results. | 1. All participants correctly interpret the results. |  |
