# Supplementary figures and images for: Ability to understand and correctly follow HIV self‐test kit instructions for use: applying the cognitive interview technique in Malawi and Zambia
Source: J Int AIDS Soc. 2019 Mar 25;22(Suppl Suppl 1):e25253. doi: 10.1002/jia2.25253 (PMC6432102; doi:10.1002/jia2.25253)

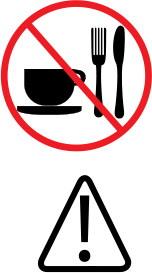


**
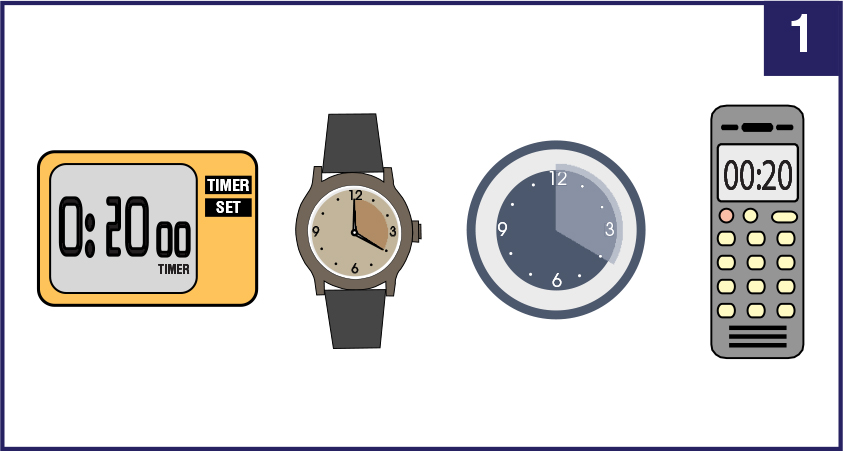
**

**
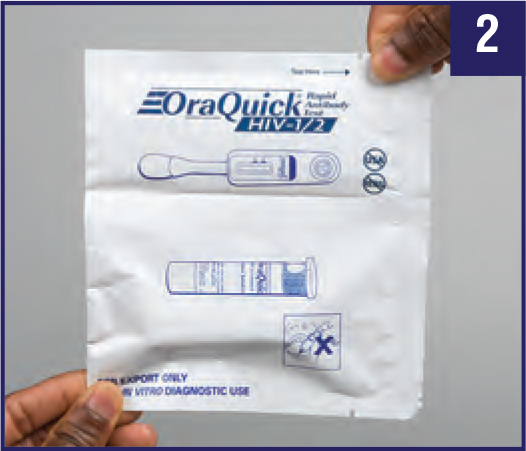
**


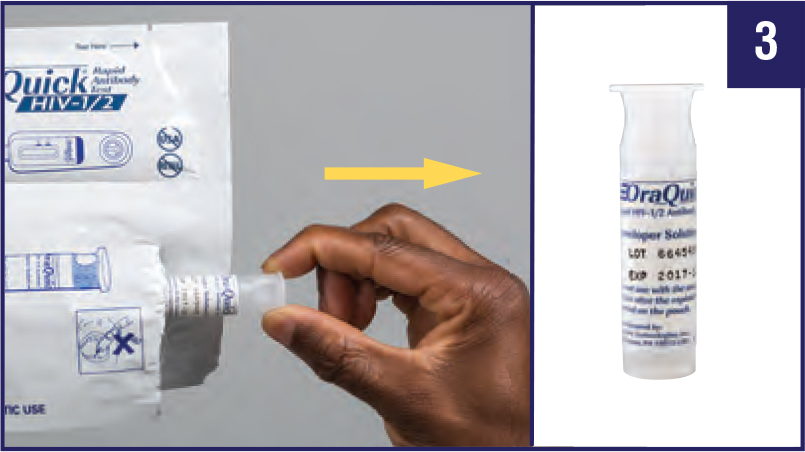


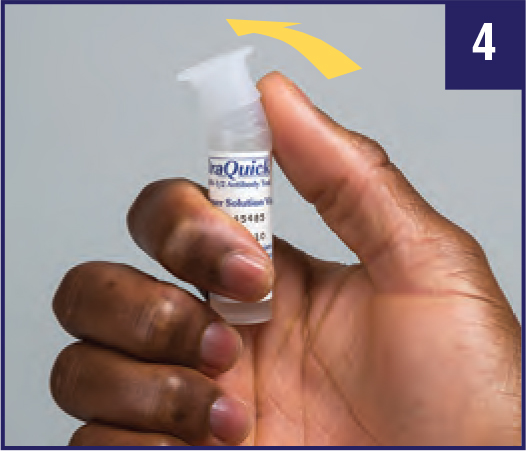


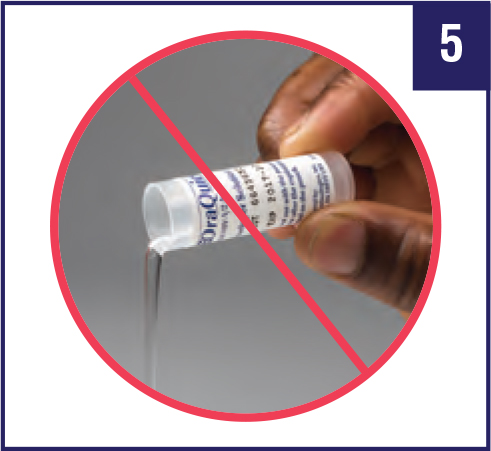


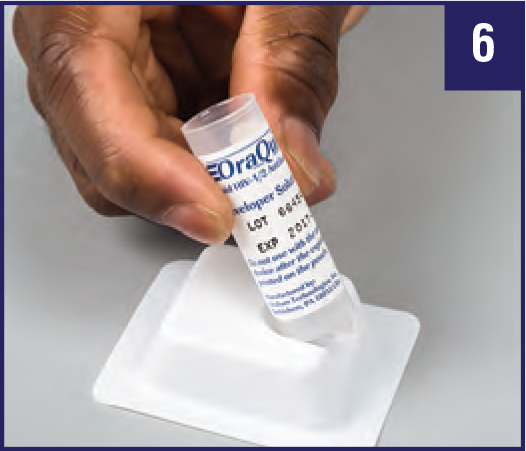


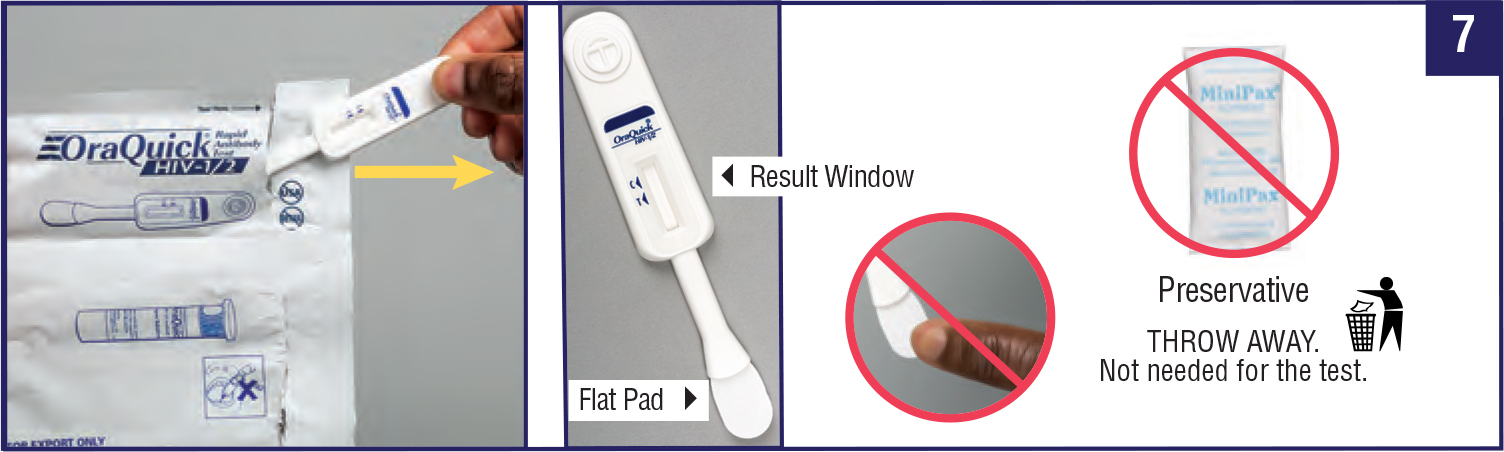


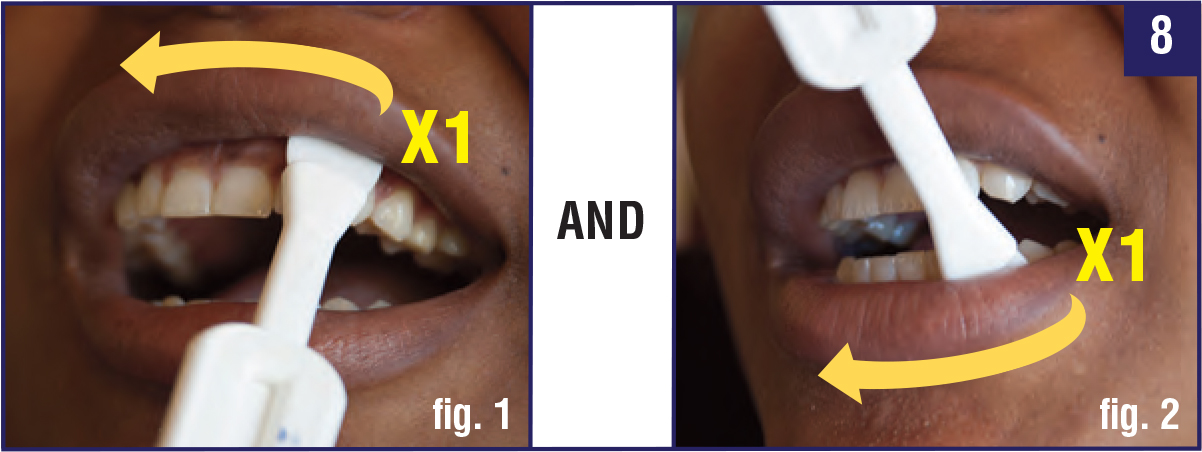


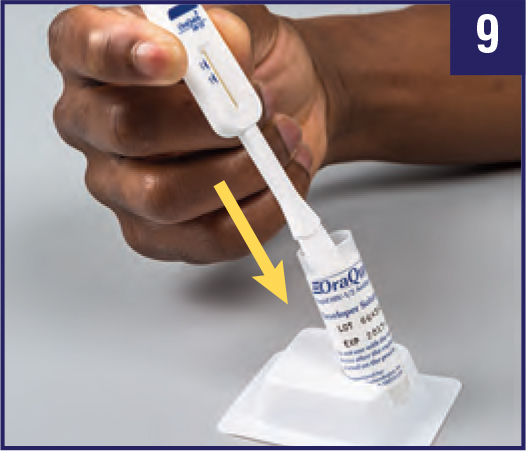


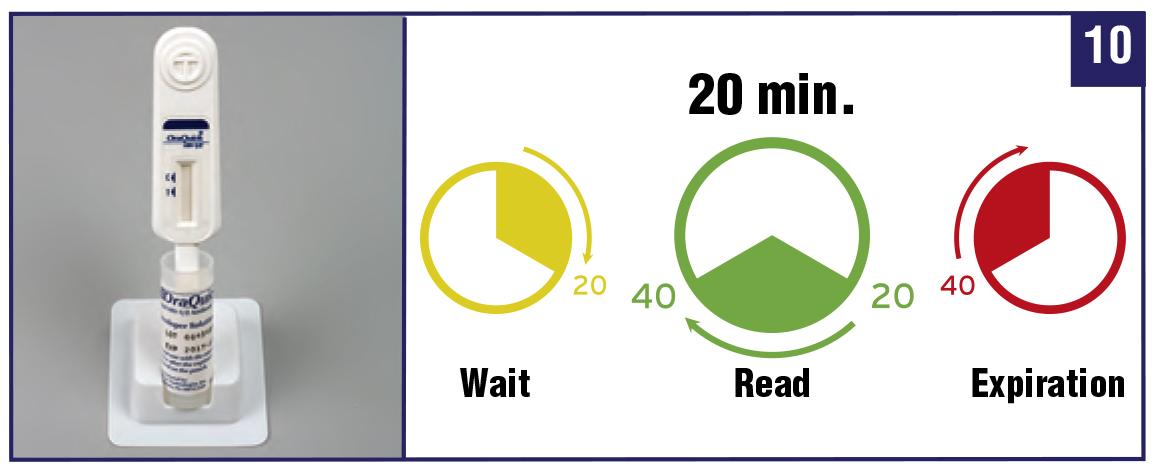


**HIV POSITIVE RESULT**

**
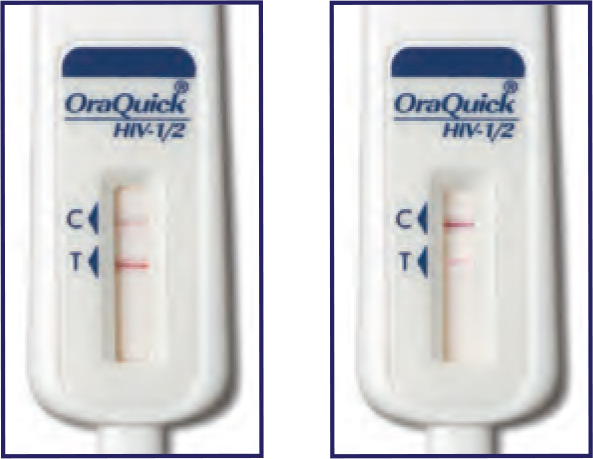
**

**HIV NEGATIVE RESULT**

**
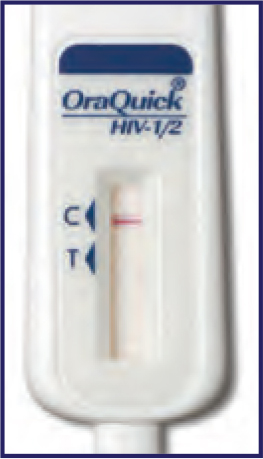
**

**INVALID RESULT**


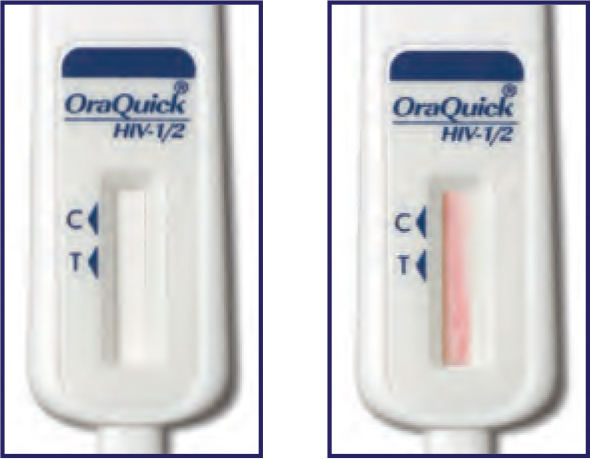


**DISPOSAL**


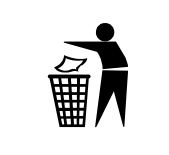

Supplement: Supplementary file 2 [file JIA2-22-e25253-s001.doc]
